# Silicon and Plant–Animal Interactions: Towards an Evolutionary Framework

**DOI:** 10.3390/plants9040430

**Published:** 2020-04-01

**Authors:** Ofir Katz

**Affiliations:** Dead Sea and Arava Science Center, Mt. Masada, Tamar Regional Council, Tamar 86910, Israel; katz.phyt@gmail.com; Tel.: +972-52-288-5563

**Keywords:** evolution, herbivory, phytoliths, plants, silicon

## Abstract

Herbivory is fundamental in ecology, being a major driver of ecosystem structure and functioning. Plant Si and phytoliths play a significant antiherbivory role, the understanding of which and of its evolutionary context will increase our understanding of this phenomenon, its origins, and its significance for past, extant, and future ecosystems. To achieve this goal, we need a superdisciplinary evolutionary framework connecting the role of Si in plant–herbivore interactions, in global processes, and in plant and herbivore evolution. To do this properly, we should acknowledge and incorporate into our work some basic facts that are too often overlooked. First, there is great taxonomic variance both in plant Si contents, forms, and roles, but also in herbivore responses, dietary preferences, and in fossil evidence. Second, species and their traits, as well as whole ecosystems, should be seen in the context of their entire evolutionary history and may therefore reflect not only adaptations to extant selective factors but also anachronistic traits. Third, evolutionary history and evolutionary transitions are complex, resulting in true and apparent asynchronisms. Fourth, evolution and ecology are multiscalar, in which various phenomena and processes act at various scales. Taking these issues into consideration will improve our ability to develop this needed theoretical framework and will bring us closer to gaining a more complete understanding of one of the most exciting and elusive phenomena in plant biology and ecology.

## 1. Introduction

Many plant species can take up silicon (Si) from the soil in the form of monosilicic acid (Si(OH)_4_), and thereafter accumulate it in their tissues in various forms; mostly monosilicic acid and amorphous silica phytoliths (Si(OH)_2_·*n*H_2_O). This capability and the accumulated amounts are a function not only of internal mechanisms (manifested by taxonomic and phylogenetic signals in plant Si contents [1,2,3]) but also of environmental factors, including water availability, transpiration, and exposure to herbivory [4,5,6]. Si accumulation has multiple benefits to accumulating plants, protecting them from a myriad of abiotic stresses [7], pathogen intrusions [8,9], and herbivory [10,11]. Consequently, Si accumulation in plants also affects other ecosystem components and functions, e.g., herbivore performance and population dynamics [10,11,12,13,14] and resource utilization and cycling [15,16,17,18]. Therefore, Si content is a plant functional trait that is involved in driving ecosystem functioning and responses to environmental change [1,2,15,19,20,21,22].

Owing to it being the second most abundant element in the Earth’s crust [23], which is accumulated in plants and thus affects ecosystem functioning and global processes, such as the carbon cycle [15,16,17], plant Si accumulation is at the interface between Earth (mainly geology/pedology, meteorology/climatology, and paleontology) and life sciences (mainly ecology, evolution, and anatomy/physiology) [19,24]. Therefore, the culminating merger of Earth and life sciences disciplines into a single Earth–life superdiscipline both offers a promising future for plant Si and phytoliths research and can draw from the experience of superdisciplinary plant Si and phytolith research [24]. The study of plant Si roles in the ecology and evolution of plant–herbivore interactions is a good example for such an integration. Herbivory is a key ecological phenomenon with great impact on evolution. Apart from very few exceptions, the minimally complex complete sustainable terrestrial ecosystem must include inorganic carbon and energy sources (primarily CO_2_ and sunlight), a chemically appropriate liquid medium (water), an autotroph (commonly plants) that converts inorganic carbon into organic compounds, and a decomposer that recycles these organic compounds back into inorganic carbon. Herbivory, i.e., primary consumption of autotroph biomass by a heterotroph, represents the most basic supplementary functions and complexity to this minimally complex complete sustainable ecosystem, through three main paths. First, herbivores exert direct top-down pressure on plants, which translates into removal of biomass, population regulation, and selective pressure [25,26,27]. Second, herbivores may assist in plant reproduction through pollination, seed dispersal, and seed germination [25,28]. Third, herbivores play a role in ecosystem metabolism and resource cycling, mostly by shortening turnover time of plant biomass [29,30,31] (for examples from the plant Si domain, see [1,32,33]). Hence, on large temporal and spatial scales, herbivory can be a major driver of plant evolution, through escalation, mutualism, or specialization, and through accelerating resource cycling and release [34].

Here, I discuss the potential for developing a superdisciplinary evolutionary framework connecting the role of Si in plant–herbivore interactions, global processes, and plant and herbivore evolution. This framework will set the infrastructure to advance our understanding of the evolution of ecosystems and the ecosphere and specifically of Si’s antiherbivory role, its origins, and its implications for current and future environmental challenges. Nevertheless, developing such a framework in a manner that allows appropriate and correct integration of knowledge requires paying attention to four major issues at the interface between paleobiology and extant biological systems. Therefore, the main goal of this manuscript is not to present a framework, but to discuss how it can be developed. The main challenge is not a lack of data and knowledge—we know a lot about Si’s role in plant–herbivore interactions and about the evolutionary history of Si and phytoliths in relation to herbivory—but how to integrate this knowledge, interpret it correctly, and plan future research aimed towards this desired framework.

## 2. Taxonomic Variances

The first thing to remember is that not all plant taxa accumulate Si to the same extent, and there are strong differences of several orders of magnitudes among plant taxa [1,2,3]. These differences also reflect differences in plant Si uptake mechanisms and forms: some species exclude Si actively, some take it up passively, and others take it up actively [35]. Monosilicic acid occurs virtually in all species that accumulate Si to any extent, but phytoliths form only in those plant taxa that accumulate Si above a certain level. In active Si accumulators, phytoliths are probably the most abundant Si form. This variance implies that different plant taxa utilize Si differently, not only in terms of its uptake but also in terms of its internal functions. In the case of herbivory, a few studies have suggested that monosilicic acid may be involved in systemic responses, but no clear connections or mechanisms could be shown [8,9], whereas phytoliths are probably mostly a physical defense [10,12]. Finally, we have increasing evidence that Si may be a partial substitute for carbon, as evidenced by tradeoffs between Si and carbon-based structural lignin and cellulose [36] and between Si and carbon-based defenses [37]. The possibility for such tradeoffs is further supported by previous studies that suggest Si utilization is energetically cheaper than utilizing carbon for comparable functions [38], and thus can play a significant role in plant Si ecology [19]. However, since plant taxa vary in Si contents, its forms, and its functions, we must also consider that Si uptake and accumulation and its tradeoffs with carbon evolved for different reasons and in different paths, and we must implement this possibility in our attempts to understand the role of Si in both plant ecology and evolution, let alone when focusing on a single role (e.g., herbivory).

Similar ideas follow for herbivores. Invertebrate, small vertebrate, and large vertebrate herbivores vary in their responses and preferences to Si. First, invertebrate and vertebrate physiology is different and so is their plant consumption mode. Invertebrates usually can only remove a small part of the leaf in each bite, sometimes leaving partially eaten leaves. Vertebrate herbivores, especially the larger ones, can remove an entire leaf in one bite, or even entire plants (mostly shallow-rooted annual species). Second, size differences usually imply differences in effects of dietary deficiency and in feeding selectivity. Larger herbivores tend to be less sensitive to low food quality and thus can be less selective. For example, the inability of locust to break down cells that are protected by Si and extract all of their chlorophyll [12] may not apply for large vertebrates, whereas small vertebrates may be affected similarly [10,11] but not necessarily to the same degree. Likewise, the damage caused by phytoliths to the esophagus is likely to be more harmful to invertebrates (sufficiently large phytoliths may even obstruct the esophagus of some very small invertebrates) but are likely to cause only superficial damage to large vertebrates’ esophagi. On a similar note, vertebrates with continually-growing, continually-replaced, or high-crowned teeth are less likely to sustain long-term damage from phytolith abrasiveness [39] (but see a critical discussion of hypsodonty and phytolith coevolution [40,41,42,43]).

Finally, any evolutionary framework must consider the differences between what we know from extant species and what we know about deep time. For example, while nearly all studies of extant plant–vertebrate herbivore systems tend to focus on mammals (reptilian herbivory is far less common), it is now evident that ancestral Si-rich grasses were also a part of herbivorous dinosaurs’ diets [44,45], and the evolution of early Si-rich angiosperms may have been directly or indirectly affected by these herbivores [1]. Furthermore, plant fossil evidence for invertebrate herbivory is relatively abundant and goes back to the earliest days of terrestrial herbivory [46], whereas evidence for plant consumption by vertebrate herbivores relies mostly on toothwear and gut/coprolite content [44,45,47]. This is mostly because of differences in plant consumption modes (see above), but also because fossil gut content and herbivore coprolites are rare and methods for their analysis are underdeveloped [48,49,50,51,52,53]. Direct fossil evidence for consumption of Si-rich plants by invertebrates is far more meagre, practically nonexistent Therefore, the rest of this manuscript will focus on mammalian and other vertebrate herbivores.

## 3. Anachronisms

“Evolutionary biologists are very fond either of pretending that the plant traits we see are selected for and maintained by current interactions or, at least, of choosing to work on those systems that seem to match this assumption. However, we all know perfectly well that a plant (and its herbivores) is a collection of anachronistic traits that at any given time have caught up with contemporary selective pressures to a highly variable degree.” (Daniel H. Janzen [54], p. 339)

This observation by Janzen puts forward a major challenge in any attempt to understand extant species’ ecology. The evolution of any one species is interlinked with the evolution of species with which it interacts (even if these interactions are not intimate or direct), as well as with changes in its physical environment. Nonetheless, such changes do not always result in evolutionary transition in that one species. Thus, we cannot assume that a species’ traits at any time of its evolutionary history necessarily reflect selective forces that acted on it at (or shortly before) that time [55,56]. Plant adaptations to herbivory are not an exception. Various plant species have traits that evolved as adaptations to herbivores with which they no longer interact, e.g., wide-angle branching against now-extinct herbivorous terrestrial birds [57,58] and spiny tree trunks against now-extinct climbing herbivorous lemurs [59]. In other cases, traits may be traced back as adaptations to herbivores that still exist in the plants’ areas of origin but not necessarily in its extant environment. For example, most spiny trees in Israel are of Saharo-Arabian or Sudanian origins and have spines protecting their vegetative (rather than reproductive) parts, whereas tree species of Mediterranean origins generally lack spines, possibly reflecting differences between folivores of African savannas and frugivores of Mediterranean maquis [60].

Si as an antiherbivory defense is no different. Si-rich horsetails dominated many parts of the world and were prominent in herbivorous dinosaurs’ diets during the Jurassic and into the Cretaceous [47,61], so their current high Si and phytolith contents [3,62]—as far as they reflect an antiherbivory role—may be anachronistic. Moreover, while we nowadays pay most of our attention to Si as an antiherbivory defense mostly among the highly Si-rich grasses, many other angiosperms also accumulate Si [1,2,3]. Although Si accumulation in these species, its variation in response to herbivory and its conformation to herbivory hypotheses (e.g., the resource availability hypothesis) is weaker than for grasses, suggesting a weaker antiherbivory role [63], the trait may have initially evolved in some of these clades in response to herbivory during the Cretaceous [1]. At a smaller timescale, it is possible that over 5000 years of human-induced intense grazing history in the Levant has caused some grasses in this region to constitutively invest in phytolith formation, hence eliminating the positive effect of extant herbivory on their Si content [63] that is commonly observed in other parts of the world [4,5,64,65] (but see [66,67]).

## 4. Asynchronisms

In addition to anachronisms, which are often observed in extant species, there is a second temporal challenge of asynchronisms, which appear in paleobiological studies and affect our interpretation of evolutionary transitions and our understanding of evolutionary history [1,34,68]. An evolutionary transition, as perceived by the extended evolutionary synthesis [69,70], consists of several short-duration changes (i.e., events): phenotypic shift, genotypic shift, and natural selection leading to phenotypic and/or genotypic abundance/frequency change and eventually to speciation (genetic isolation of genotypes). While all these changes are interrelated, their temporal order and the temporal distances among them can vary greatly [69]. Therefore, the chronology of evolutionary transitions depends on the event that is dated and the dating method [68]. Fossils can indicate trait emergence, and their abundance can indicate “success” and thus possibly selection in favor of a trait. Molecular methods can date genotype emergence and genetic isolation, which themselves are indicated by different gene sequences (trait-coding sequences vs. noncoding or organellar sequences). Therefore, we can have various asynchronous dates for the same evolutionary transition. One of the most striking examples is that although plastid DNA phylogenies date the gymnosperm-angiosperm divergence to 300–350 Ma, the oldest angiosperm-like traits have been observed only 150–200 Ma [34,71]. The need to resolve this extraordinary asynchronism—a gap spanning over one quarter to one half of the entire history of terrestrial life (!)—opens many new questions about plant evolution and about evolutionary theory altogether [34,68,71].

Asynchronisms are not strangers to the evolution of plant Si uptake and accumulation. Trembath-Reichert et al. [72] carried out a thorough phylogenetic analysis of plant Si transporters and traced their origins as far as 400 MA back. While this is fascinating evidence that greatly advances our understanding of plant Si’s molecular evolution, its implications for the evolution of Si uptake and for plant–herbivore interactions are limited. Because multiple transporters are involved in Si uptake and accumulation, this phylogenetic analysis does not provide clear information concerning what plant groups possessed this trait and when, and when the trait (rather than the transporter, i.e., phenotype over genotype) emerged within plant evolutionary history [68]. A second example is the central role of plant Si and phytoliths as an antiherbivory defense in the study of the Neogene Revolution, in which forests gave way to grasslands [73,74]. The dominant paradigm during most of the second half of the 20th century was that these grass- and grazer-dominated ecosystems formed through coevolutionary escalation between phytolith-rich grasses and large mammalian herbivores possessing abrasion-adapted dentition [39,75]. However, this paradigm is nowadays challenged, in part because the emergence of grass-dominated vegetation and abrasion-adapted dentition is asynchronous: the former predates the latter by several million years in North America and Eurasia [40,41,73,76], but the latter predates the former by several million years in South America [42,43]. Furthermore, phylogenetic dating of the emergence of Si-rich angiosperm clades is asynchronous with putative environmental drivers, such as herbivory and atmospheric CO_2_ decline [77].

Nevertheless, this last study [63] searched for coalescences of molecular (genetic emergence) times and paleontological (trait “success”) times, which represent two different parts of evolutionary transitions that are prone to be asynchronous. An attempt to overcome this asynchronism can be found in another phylogenetic analysis, which attempts to estimate trait “success” times from clade emergence times. The emergence of angiosperm orders with Si-rich species has peaked at around 113–100 Ma, suggesting that the majority of Si-rich clade diversification took place later, approximately 85 Ma, the same time when abrasion-adapted dentition evolved in herbivorous hadrosaurs, ceratopsians, and gondwanatherians [1]. However, even in this case, Si-rich species’ “rise to success” may not have been a case of direct plant–herbivore coevolution. Rather, herbivory of Si-rich horsetails could have merely supplied ample available Si for early Si-accumulating angiosperms to use for other purposes [1]. While direct evidence for Si-rich species’ “success” during that time is missing, leaving this coalescence hypothetical, the inclusion of ancestral grass phytoliths in hadrosaur dentition from 113–110 Ma [44] and in titanosaur coprolites from 67 Ma [45] suggests that herbivorous dinosaurs did consume ancestral Si-rich grasses and angiosperms and may have been a selective pressure in favor of this trait within that timeframe.

## 5. Scales

Anachronisms and asynchronisms mainly involve macroevolution at supra-millennial temporal scales. However, a complete evolutionary framework should not ignore microevolution or macroevolution at smaller temporal (and spatial) scales. Differences among such temporal and spatial scales, and how to resolve them, posit another challenge [78], to a large extent because the environmental drivers of evolution vary among scales. At relatively small temporal and spatial scales, evolution is driven mainly by biotic interactions (Red Queen model), whereas at larger temporal and spatial scales evolution is driven mainly by abiotic factors (Court Jester model) [79]. Moreover, identifying Red Queen and Court Jester processes in deep time and distinguishing between them is challenging, in part because the ability to identify these processes depends on temporal resolution and on the spatial scale at which analyses are carried out [79,80,81,82,83] (and see also [84]). Although Red Queen processes are usually more important than Court Jester processes at smaller scales, there is no clear scalar dichotomy between the two. Court Jester processes can be significant even at relatively small scales, and Red Queen processes can be significant in relatively large scales.

Studies of herbivory effects on plant Si uptake and accumulation offer a simple example. Effects of water availability and herbivory on plant Si uptake and accumulation are more often seen and tend to be greater in controlled laboratory experiments than in field studies of naturally-growing plants [4,66]. This disparity occurs, at least in part, because naturally-growing plants are exposed to more variable sets of environmental (abiotic and biotic) conditions, whose effects on Si uptake and accumulation can confound those of a single laboratory-controlled variable. For example, the effects of herbivory on Si uptake and accumulation can be masked, in comparative studies of naturally-growing plants in different ecosystems, by climate [4,63], soil water availability [4,63,66], soil nutrient availability [66], and grazing history [63,66]. Moreover, intense millennial-scale grazing on certain species may cause them to have constituently high Si contents [63], suggesting selection in favor and fixation of high Si contents in time scales of—at least—generations to millennia.

Hence, studies at different temporal and/or spatial scales should consider differences among scales and scales’ correspondences to the Red Queen and Court Jester models [78,79]. Therefore, the essence of the scale challenge in the development of any evolutionary framework can be summarized to three key questions. First, what selective pressures or other forces act at various scales (e.g., temporal, spatial, ecological, and micro/macroevolutionary)? Second, whether and how can we interlink selective pressures and processes at various scales into a coherent multiscalar theory? Third, whether and how can we scale up and down to overcome cases where such interlinks are missing?

For example, the discourse over the Neogene Revolution [73,74,77] and the earlier origins of Si accumulation in angiosperms [1] discusses both abiotic and biotic forcings, but seldom consider scale differences within and among these studies, or that abiotic and biotic forcings possibly operate at different scales. Likewise, in ecological studies of plant–herbivore interactions, controlled experiments at smaller scales can yield results that are significantly different than those obtained in naturally-growing organisms, the latter usually reflecting larger scales [4,63,66]. As stated above, this scale disparity is often implicit rather than explicit. Micro and macroevolution inherently reflect different scales but are both driven by processes at multiple and often overlapping scales. Therefore, scale differences need to be explicit and accounted for in any evolutionary study, let alone in the development of evolutionary frameworks that are inherently multiscalar.

Lastly, we should bear in mind that we infer the biology, ecology, and evolution of past organisms and ecosystems based on our knowledge and understanding of the biology, ecology, and evolution of extant organisms and ecosystems, but at the same time these extant organisms and ecosystems are (anachronistic) products of past organisms and ecosystems. Of course, paleobiologists and ecologists are very well aware of this chicken-and-egg problem and accordingly are cautious in their inferences. Ecological processes and patterns have evolved over the Phanerozoic [85,86,87,88], and likewise herbivory has also evolved and become more complex over geologic time [46,89], apparently with important consequences for plant evolutionary patterns [34]. Thus, it cannot be assumed that ecological processes and evolutionary forcings are unchanged throughout geologic time, and any evolutionary framework that refers to this temporal scale should consider an evolution of ecological phenomena and of ecology itself. With evidence for the evolution of herbivory and plant–herbivore interactions, let alone for its implications for plant evolution [34,46,89], the proposed evolutionary framework discussed in this manuscript must take the evolution of ecology into consideration.

## 6. Towards an Evolutionary Framework

Herbivory is fundamental in almost all ecosystems and is a major driver of ecosystem structure and functioning. There is currently ample and growing evidence that plant Si and phytoliths play an antiherbivory role in many plant species. Therefore, understanding the antiherbivory role of Si and phytoliths and its evolutionary context is essential for our understanding of the evolutionary history and ecology of plants, herbivores, ecosystems, and the Earth–life interface. Gaining this knowledge relies in part on developing a superdisciplinary evolutionary framework connecting the role of Si in plant–herbivore interactions, global processes, and plant and herbivore evolution. Nevertheless, developing such a theoretical framework and correct interpretation of knowledge within it requires that we understand, consider, and properly handle several issues. Specifically, we need to consider taxonomic variances in plant accumulation, forms, functions, and ecology, as well as taxonomic variances in herbivore feeding and responses. We need to remember that species, traits, and ecosystems have their own evolutionary histories, so any trait or state should not be assumed to reflect adaptation to extant selective forcing but may reflect historical and obsolete conditions. Evolutionary history and evolutionary transitions are complex, so care should be given to what is dated and what the dating actually means. Finally, the multiscalarity of evolution and ecology means that it is important to understand at which scale(s) a study is carried out and whether and how gained knowledge can be upscaled or downscaled, and how to create an appropriate multiscalar understanding.

These issues need to be embedded within the attempt to develop a framework in two complementary manners. First, we should be more critical in our treatment of existing data and knowledge, asking what parts of it may be misinterpreted or incomplete. Second, we should fill in any gaps that may cause data misinterpretation. This means not only filling specific gaps we may find in current data and knowledge (e.g., new dating to avoid asynchronisms) but also to devise new methods to make the best of existing and potential evidence (e.g., improve methodologies for studying herbivore diets in deep time). Most importantly, the design of future research programs should consider the issues raised in this manuscript. Big theoretical questions like the role of Si in the ecology and evolution of plant–herbivore interactions require well-structured theoretical frameworks that integrate many lines of evidence from multiple disciplines. It is more appropriate to design research that inherently aims at such integrations than to patch up knowledge post-hoc. I am certain that embedding these ideas into our thinking will enable us to gain a better understanding of the biology, ecology, and evolution of one of plants’ most exciting and elusive traits.
